# Volatiles Emitted at Different Flowering Stages of *Jasminum sambac* and Expression of Genes Related to α-Farnesene Biosynthesis

**DOI:** 10.3390/molecules22040546

**Published:** 2017-03-29

**Authors:** Ying Yu, Shiheng Lyu, Dan Chen, Yi Lin, Jianjun Chen, Guixin Chen, Naixing Ye

**Affiliations:** 1 College of Horticulture, Key Laboratory of Tea Science, Fujian Agriculture and Forestry University, Fuzhou 350002, Fujian, China; 1130311016@fafu.edu.cn (Y.Y.); 2140305002@fafu.edu.cn (S.L.); 1150311002@fafu.edu.cn (D.C.); 152331405@st.usst.edu.cn (Y.L.); 2 Department of Environmental Horticulrture and Mid-Florida Research and Education Center, University of Florida, IFAS, Apopka, FL 32703, USA

**Keywords:** jasmine, *Jasminum sambac* (L.) Aiton, α-farnesene, 3-hydroxy-3-methylglutaryl coenzyme A synthase (HMGS), 3-hydroxy-3-methylglutaryl coenzyme A reductase (HMGR), farnesyl pyrophosphate synthase (FPPS), lovastatin, terpene synthase (TPS)

## Abstract

Fresh jasmine flowers have been used to make jasmine teas in China, but there has been no complete information about volatile organic compound emissions in relation to flower developmental stages and no science-based knowledge about which floral stage should be used for the infusion. This study monitored volatile organic compounds emitted from living flowers of *Jasminum sambac* (L.) Ait. ‘Bifoliatum’ at five developmental stages and also from excised flowers. Among the compounds identified, α-farnesene, linalool, and benzyl acetate were most abundant. Since α-farnesene is synthesized through the Mevalonate pathway, four genes encoding 3-hydroxy-3-methylglutaryl coenzyme A synthase, 3-hydroxy-3-methylglutaryl coenzyme A reductase (HMGR), farnesyl pyrophosphate synthase, and terpene synthase were isolated. Their expression patterns in living flowers at the five stages and in excised flowers coincided with the emission patterns of α-farnesene. Application of lovastatin, a HMGR inhibitor, significantly reduced the expression of the genes and greatly decreased the emission of α-farnesene. The sweet scent was diminished from lovastatin-treated flowers as well. These results indicate that α-farnesene is an important compound emitted from jasmine flowers, and its emission patterns suggest that flowers at the opening stage or flower buds 8 h after excision should be used for the infusion of tea leaves.

## 1. Introduction

*Jasminum sambac* (L.) Ait., commonly known as jasmine, is a member of the olive family (Oleaceae) and native to India [1]. It is a small shrub or vine growing up to 3 m in height that is fairly hardy and drought-resistant. Jasmine flowers are sweetly fragrant and are used for the preparation of an essential oil and for making jasmine tea. Jasmine oil has a wide range of medicinal applications and can be used in the perfumery, soaps, flavorings, and the cosmetic industries [2]. Jasmine teas are produced when fine-quality tea leaves are scented with fresh jasmine blossoms [3,4]. Jasmine flowers have also been used as a drug for the treatment of diarrhea, abdominal pain, conjunctivitis, and dermatitis [5].

To produce high quality jasmine tea, in addition to the quality of tea leaves used as its base, the quality of jasmine flowers are critically important. Quality means not only the appearance and freshness of the flowers but also the developmental stage of flowers in emission of fragrance. Jasmine flowers bloom for only a day, lasting for 12 to 20 h. Flowers open at night, usually around 6 to 8 p.m. [6]. Jasmine producers usually pluck flower buds during the day. The plucked flower buds are not stored but are directly delivered to tea factories for the scenting process during that night. Plucking jasmine flowers requires experience to ensure that the plucked buds will bloom that night. Buds that have already opened are not usable; and plucked buds that do not open that night are not useful either. This harvesting practice has endured for more than 1000 years in China. However, questions as to the dynamics of aroma emission in relation to flower developmental stages, particular compounds emitted relevant to enrich aroma in jasmine tea, and the appropriate time for harvesting flowers for either immediate use or short-distance transport have not been well studied.

Floral scent volatiles generally fall into the terpenoid or phenylpropanoid/benzenoid classes [7]. Ragasa [8] isolated caryophyllene oxide, a mixture of benzyl benzoate and farnesyl acetate, methyl isoeugenol, squalene, and sitosterol from *J. sambac* flowers and suggested that benzyl benzoate, farnesyl acetate, and methyl isoeugenol were responsible for the flower scent. However, Pragadheesh et al. [9] identified a total of 23 volatile organic compounds from *J. sambac* flowers and reported that the major proportion of the fragrance was comprised of *cis*-3-hexenyl acetate, (*E*)-β-ocimene, linalool, benzyl acetate, and (*E*,*E*)-α-farnesene, of which (*E*,*E*)-α-farnesene (C_15_H_24_, M.W. 204) accounts for 80%. Bera et al. [10] identified 49 compounds in flowers of four species of *Jasminum* where linalool and α-farnesene were the major monoterpene and sesquiterpene in jasmine flowers, and benzyl acetate was the most abundant benzenoid. The authors further indicated that floral scent of *J. sambac* includes three major benzenoid esters: benzyl acetate, methyl benzoate, and methyl salicylate, and three major terpene compounds: (*E*)-ocimene, linalool, and α-farnesene.

In addition to the variable results about major volatiles emitted from flowers of *J. sambac*, compounds that contribute to sweet scent in jasmine tea have not been well elucidated. Ito et al. [11] analyzed Chinese jasmine green tea scented with flowers of *J. sambac* and found that main volatile compounds included (*Z*)-3-hexenol, linalool, benzyl alcohol, benzyl acetate, methyl anthranilate, and indole. Lim [5] suggested that methyl anthranilate and linalool were the key odorants of the jasmine tea flavor. Recently, Lin et al. [12] reported that the main compounds in jasmine teas were α-farnesene, (*Z*)-3-hexenyl benzonate, linalool, benzyl alcohol, benzyl acetate, methyl anthranilate, and indole. The results of Lin et al. [12] and Ito et al. [11] are similar except for α-farnesene that was missed in the report of Ito et al. [11]. As stated by Lin et al. [12], the inconsistance could be due to the different extraction and sample preparation methods. Additionally, the freshness of jasmine teas used for analysis is important. Jamsine teas may deteriorate quickly if they are stored around 24 °C. To maintain their flavor and freshness, jasmine teas are commonly well sealed and stored at low temperatures [13].

To determine the release patterns of volatile organic compounds in relation to flower development, this study analyzed major volatile organic compounds emitted from flowers at five developmental stages and also at different time after flower bud excision, identified α-farnesene as one of important compounds released from both living and excised flowers, isolate and characterize genes associated with α-farnesene biosynthesis, and determine their expression levels from flower opening to senescence relevant to α-farnesene emission. The information gathered from this study could aid in using developmentally appropriate flowers for processing quality jasmine teas.

## 2. Results

### 2.1. Flower Developmental Stages and Major Volatile Compound Emissions

**Figure 1:**
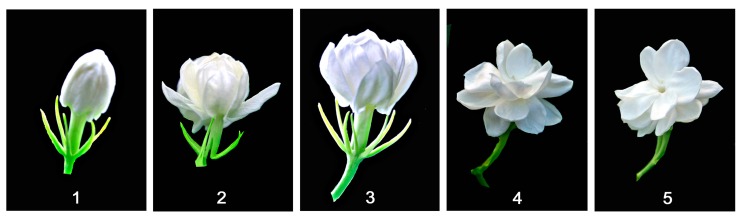
Five flowering stages of *J. sambac* ‘Bifoliatum’: (**1**) Flower at closed bud stage; (**2**) flower starts opening; (**3**) flower opening; (**4**) fully opened flower, and (**5**) flower starts senescence.

Flowers of *J. sambac* ‘Bifoliatum’ were divided into five developmental stages: (1) Bud stage where all petals were close together; (2) initial-flowering stage, i.e., outside petals started reflex outwardly; (3) flower opening stage in which all petals became reflex and were ready to fully open; (4) flower fully opening stage, all petals completely opened with half of the petals becoming droopy; and (5) initial senescence stage, i.e., petals start withering (Figure 1).

The GC-MS analysis showed that a total of 30 volatile organic compounds were emitted from flowers of *J. sambac* ‘Bifoliatum’. Table 1 presents 13 major compounds (higher chromatographic areas) emitted from living flowers at the five developmental stages. The concentrations were determined relative to the internal control of ethyl decanoate except for α-farnesene which was calculated based on standard curve. Compounds released from closed buds were low except for linalool, a monoterpene, which was 3.89 mg/kg FW (fresh weight). Linalool drastically increased to 41.86 mg/kg FW at stage 2, then dropped to about 22 mg/kg FW at stages 3 and 4, and further decreased to 8.38 mg/kg FW. On the other hand, α-farnesene, a sesquiterpene was detected at stage 2 and increased from 12.08 mg/kg FW to 53.86 mg/kg FW at stage 3, then decreased to 39.03 mg/kg FW at stage 4 and 20.71 at stage 5. Another compound, benzyl acetate exhibited the same emission pattern as α-farnesene but at much lower concentration than α-farnesene. The rest compounds had concentrations much lower than the previously mentioned ones.

The 13 compounds identified from living flowers were also the major compounds emitted from excised flowers (Table 2). Linalool emission was 15.76 mg/kg FW 2 h after excision, increased to 19.52 at 4 h, 22.03 at 8 h, and decreased to 12.66 mg/kg FW at 16 h after excision. The emission of α-farnesene increased from 1.45 mg/kg FW at 2 h after excision to 12.20 mg/kg FW at 4 h, 30.66 mg/kg FW at 8 h and sustained 30.71 mg/kg FW at 16 h after excision. Emitted benzyl acetate was low after excision to 2 h later, but its emission increased to 3.69 mg/kg FW at 4 h, 16.68 mg/kg FW at 8 h, and then decreased to 12.66 mg/kg FW at 16 h. Similar to the living flowers, other compounds in excised flowers were lower.

### 2.2. Analysis of Key Genes Related to α-Farnesene Biosynthesis

Results from the analysis of both living flowers and excised flowers suggested that (*E*,*E*)-α-farnesene was the predominant volatile compound emitted from *J. sambac* ‘Bifoliatum’. The biosynthesis of α-farnesene occurs through an isoprenoid pathway, commonly known as Mevalonate pathway (MVP) [14]. The α-farnesene biosynthesis uses acetyl CoA as precursor; hydroxymethylglutaric acid (HMG), mevalonic acid, and farnesyl pyrophosphate (FPP) are important intermediates [14,15,16].

**Figure 2:**
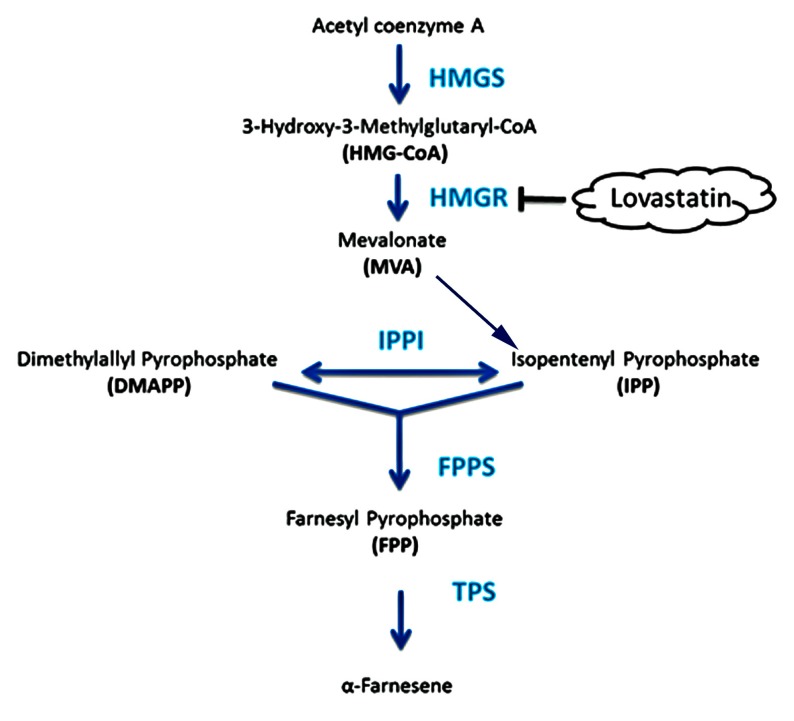
Proposed biosynthesis of α-farnesene in *J. sambac* ‘Bifoliatum’ where major enzymes include 3-hydroxy-3-methylglutaryl coenzyme A synthase (HMGS), 3-hydroxy-3-methylglutaryl coenzyme A reductase (HMGR), farnesyl pyrophosphate synthase (FPPS), and terpene synthase (TPS). Lovastatin is an inhibitor to HMGR.

As shown in Figure 2, 3-hydroxy-3-methylglutaryl coenzyme A synthase (HMGS, EC 2.3.3.10) catalyzed acetyl coenzyme A to form 3-hydroxy-3-methylglutaryl coenzyme A. 3-hydroxy-3-methylglutaryl coenzyme A reductase (HMGR, AIY26014.1) is a rate-limiting step in the pathway [17], and its activity was positively correlated with terpenoid biosynthesis [18,19,20,21,22,23]. Farnesyl pyrophosphate synthase (FPPS, AIY24422.1), another key enzyme of MVP for terpene metabolism in plants, catalyzes sequential condensation reactions of dimethylallyl pyrophosphate (DMAPP) with two units of 3-isopentenyl pyrophosphate (IPP) to form 15 carbon farnesyl pyrophosphate (FPP) [24]. Sesquiterpene synthases have been found to use the C15-diphosphate intermediate (*E*,*E*)-farnesyl diphosphate (FPP) [25]. Terpene synthase (TPS, EC 4.2.3.20) is responsible for the synthesis of the various terpene molecules from two isomeric 5-carbon precursor ‘building blocks’, leading to 5-carbon isoprene, 10-carbon monoterpenes, 15-carbon sesquiterpenes, and 20-carbon diterpenes, including α-farnesene. Thus, an attempt was made to isolate *HMGS*, *HMGR*, *FPPS*, and *TPS* from *J. sambac* ‘Bifoliatum’.

Expressed sequence tags (ESTs) for *HMGS*, *HMGR*, *FPPS*, and *TPS* were isolated from a cDNA library constructed using flowers of *J. sambac* ‘Bifoliatum’. The flank sequences of ESTs were amplified by RACE (rapid-amplification of cDNA ends) PCR to obtain the full length cDNA. GenBank accession numbers for *JsHMGR* and *JsFPPS* are AIY26014.1 and AIY24422.1, respectively.

**Figure 3:**
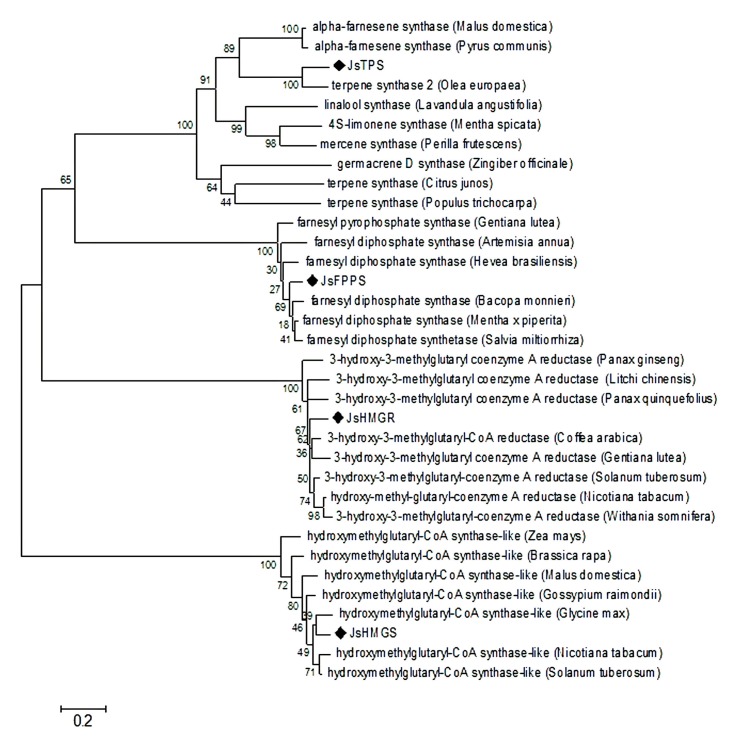
Neighbor-joining phylogenetic tree based on *JsHMGS*, *JsHMGR*, *JsFPPS*, and *JsTPS* sequence isolated from *J. sambac* ‘Bifoliatum’ in relation to respective sequences of other plant species derived from GenBank. Numerical values above the branches indicate bootstrap percentiles from 1000 replicates. The scale bar indicates the branch length that corresponds to the number of substitutions per amino acid position.

The isolated full-length cDNA of *JsHMGS* has 1808 bp and contains a 1389 bp open reading frame (ORF) (Table S1). The deduced amino acid sequence of the *JsHMGS* shares 70% to 85% similarity with *Zea mays* L., *Brassica rapa* L., *Malus domestica* Mill., *Gossypium raimondii* L., *Glycine max* (L.) Merr., *Nicotiana tabacum* L., and *Solanum tuberosum* L. (Figure 3). Phylogenetic analysis showed that *JsHMGS* was much closer to *HGMS* from *Glycine max* than other homologue genes.

The full-length cDNA of *JsHMGR* is 2274 bp long with 1785 bp ORF. The deduced amino acid sequence of the JsHMGR share 78% to 82% homolog with *Panax ginseng* C.A.Mey., *Litchi chinensis* Sonn., *Panax quinquefolius* L., *Coffea arabica* L., *Gentiana lutea* L., *Solanum tuberosum*, *Nicotiana tabacum*, and *Withania somnifera* L. (Figure 3). Phylogenetic analysis showed that JsHMGR was rather independent from the homologs in the other species but more closely related to those from *Nicotiana tabacum*, *Withania somnifera*, *Solanum tuberosum*, *Coffea arabica*, and *Gentiana lutea* (Figure 3).

The cloned full-length cDNA of *JsFPPS* is 1394 bp long and contains a 1050 bp ORF. The deduced amino acid sequence of the JsFPPS shares 85%–94% homolog with *Gentiana lutea*, *Artemisia annua* L., *Hevea brasiliensis* Mull. Arg., *Bacopa monnieri* (L.) Pennerll, *Mentha* x *piperita* L., and *Salvia miltiorrhiza* Bunge (Figure 3). *JsFPPS* is more closely associated with *Bacopa monnieri*, *Mentha* x *piperita*, and *Salvia miltiorrhiza*.

The full-length cDNA of *JsTPS* is 1884 bp long and contains a 1491 bp ORF (Table S2). The deduced amino acid sequence of the *JsTPS* shares 49%–75% homology with *Malus domestica*, *Pyrus communis* L., *Olea europaea* L., *Lavandula angustifolia* Mill., *Mentha spicata* L., *Perilla frutescens* (L.) Britton, *Zingiber offcinale* Roscoe. *Citrus junos* Siebold ex Tanaka, and *Populus trichocarpa* Torr. & A. Gray ex. Hook. Phylogenetic analysis showed that *JsTPS* was much closer to *TPS* from *Olea europaea* and also closer to *TPS* from *Malus domestica* and *Pyrus communis* than from the other mentioned species (Figure 3).

### 2.3. Relative Expression of Genes Related to α-Farnesene Biosynthesis

**Figure 4:**
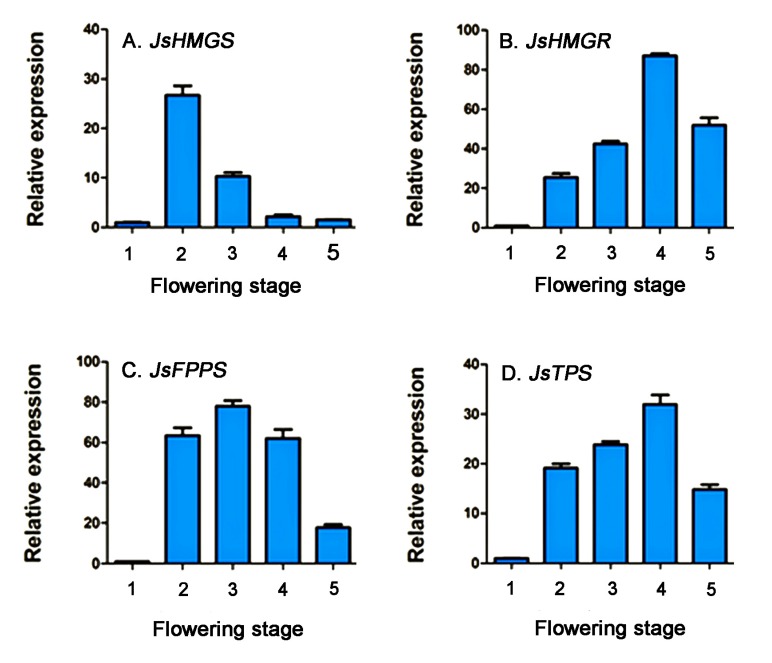
Relative expression of *JsHMGS* (**A**); *JsHMGR* (**B**); *JsFPPS* (**C**); and *JsTPS* (**D**) genes in living flowers of *J. sambac* ‘Bifoliatum’ at five flowering stages analyzed using qRT-PCR. The expression levels were normalized based on the expression of internal control gene actin and corresponding genes expressed at the flower bud stage. The bars represent standard errors of three replicates (*n* = 3).

**Figure 5:**
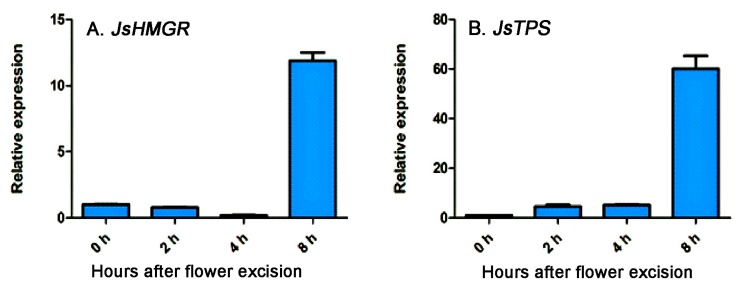
Relative expression of *JsHMGR* (**A**) and *JsTPS* (**B**) genes in *J. sambac* ‘Bifoliatum’ flowers immediately after excision to 8 h after excision analyzed using qRT-PCR. The expression levels were normalized based on the expression of internal control gene actin and corresponding genes expressed at the flower bud stage. The bars represent standard errors of three replicates (*n* = 3).

The expression patterns of four genes involving α-farnesene biosynthesis at the five flower developmental stages are present in Figure 4. *JsHMGS* expression was low at the flower bud stage and drastically increased at stage 2 and then decreased from stages 3 to 5 (Figure 4A). The expression of *JsHMGR* was also low at the bud stage, gradually increased at stage 2, peaked at stage 4, and then decreased (Figure 4B). *JsFPPS* expression was higher at stage 2, peaked at stage 3, slightly reduced at stage 4, and dramatically reduced at stage 5 (Figure 4C). The expression pattern of *JsTPS* was similar to *JsHMGR*, increased from stage 2 to 4 and decreased at stage 5 (Figure 4D). The analysis of *JsHMGR* and *JsTPS* in excised flowers showed lower expressions after flowers were excised; however, their expressions sharply increased at 8 h after excision (Figure 5).

### 2.4. Effects of Lovastatin on Gene Expression and Volatile Compound Emission

**Figure 6:**
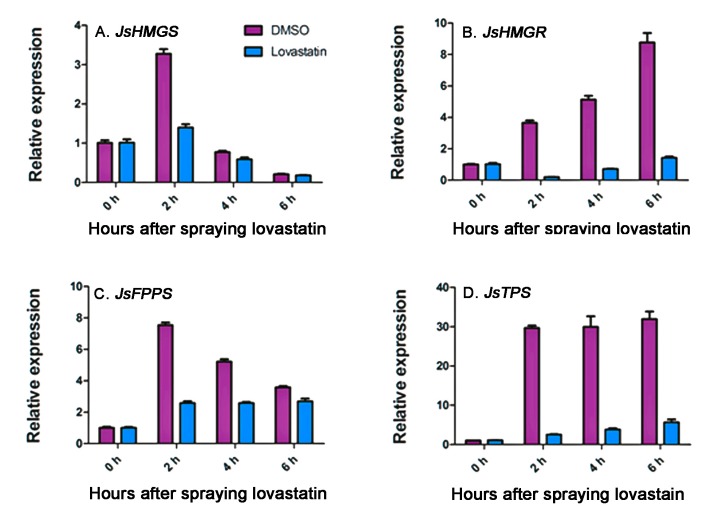
qRT-PCR analysis of *JsHMGS* (**A**); *JsHMGR* (**B**); *JsFPPS* (**C**); and *JsTPS* (**D**) expressions in *J. sambac* ‘Bifoliatum’ flowers immediately after being sprayed with 50 μm/L lovastatin or dimethyl sulfoxide (DMSO) to 6 h, thereafter. The expression levels were normalized based on the expression of the internal control gene actin and corresponding genes expressed at the bud stage. The bars represent standard errors of three replicates (*n* = 3).

Time-course expressions of *JsHMGS*, *JsHMGR*, *JsFPPS*, and *JsTPS* in flowers sprayed with lovastatin were compared to those without lovastatin treatment. The spraying signficantly reduced *JsHMGS* expression 2 h after spraying (Figure 6A) and also decreased *JsFPPS* expression from 2 to 6 h after spraying (Figure 6C). The most significant reduction happened in *JsHMGR* and *JsTPS* expression, and the suppression sustained from 2 to 6 h after lovastatin application (Figure 6B,D).

**Figure 7:**
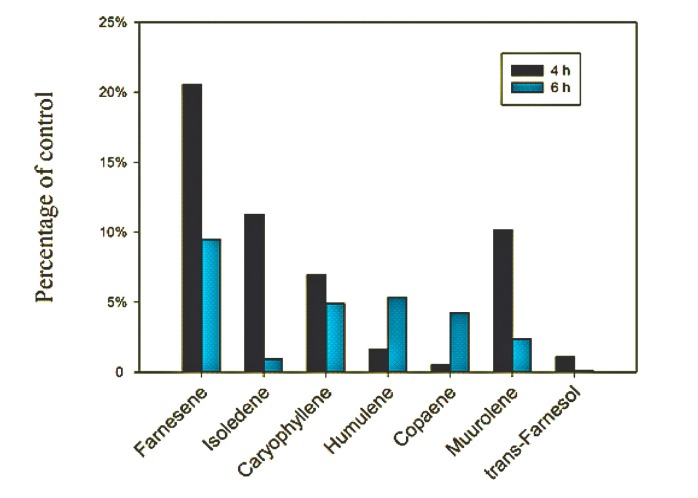
The percent reduction in emission of selected volatile organic compounds from flowers of *J. sambac* ‘Bifoliatum’ sprayed with 50 μm/L lovastatin.

The suppression of α-farnesene biosynthesis related gene expression also resulted in significant reduction in biosynthesis of volatile compounds including α-farnesene, isoledene, caryophyllene, humulene, copaene, muurolene, and trans-farnesene (Figure 7). The reductions in α-farnesene, isoledene, muurolene, and trans-farnesene were not just at 4 h but also 6 h after lovastatin application. In fact, the sweet fragance was diminished from flowers 2 h after lovastatin-application.

## 3. Discussion

*Jasminum sambac* is the primary species used in China to produce jasmine tea. This study analyzed volatile organic compounds emitted from flowers of *J. sambac* ‘Bifoliatum’ at five developmental stages, and also from excised flowers from the time of excision to 16 h thereafter. The main compounds from both living flowers and excised flowers were α-farnesene, linalool, and benzyl acetate, of which α-farnesene was the highest, followed by linalool and then benzyl acetate. Results from this study agree with the report of Bera et al. [26] that α-farnesene and linalool were the major sesquiterpene and monoterpene in flowers of *Jasminum* species and the most abundant benzenoid was benzyl acetate. However, our data showed that benzyl acetate concentrations were much lower compared to those reported by Bera et al. [10,26]. The differences may be attributed to cultivar differences as *J. sambac* cultivars produced in Fujian, China are those selected for their high sweet fragrance and used in processing jasmine teas.

Since α-farnesene is the main compound in the investigated cultivar, this study further isolated four genes *JsHMGS*, *JsHMGR*, *JsFPPS*, and *JsTPS* in the MVP pathway and analyzed their expression at the five flowering stages as well as *JsHMGR* and *JsTPS* in excised flowers. The lowest expressions of four genes at flower bud stage or initial flower excision corresponded to the lowest emission of volatile compounds. The highest expression of *JsHMGS* at stage 2 of living flowers suggested the activation of MVP; the increased expression of *JsHMGR*, *JsFPPS*, and *JsTPS* in living flowers at stages 3 or 4 and *JsHMGR* and *JsTPS* in detached flowers 8 h after excision coincided with the highest emission of α-farnesene at these stages or the time after flower excision. The results support that α-farnesene in *J. sambac* is synthesized through the MVP as shown in Figure 2, and its biosynthesis and emission are closely correlated with to the expression levels of those genes.

To further determine if the sweet fragrance of *J. sambac* is more related to α-farnesene, we sprayed lovastatin onto flowers. Lovastatin is a HMGR inhibitor, and HMGR has been considered the rate-limiting enzyme [27]. Our resulted showed that application of lovastatin significantly reduced the expression of *JsHMGS*, *JsHMGR*, *JsFPPS*, and *JsTPS*, particularly *JsHMGR* and *JsTPS*, and such reduced expressions were well correlated with the reduction of emission of seven compounds, including α-farnesene. Meanwhile, the sweet fragrance of lovastatin-treated flowers was diminished. As mentioned above, *J. sambac* flowers have been documented to emit a large number of volatile compounds belonging to terpenoids and phenylpropanoids [5,10,26]. However, no reports thus far have specifically pointed out that the sweet fragrance is mainly related to α-farnesene. Lim [5] indicated that methyl anthranilate and linalool were the key odorants of the jasmine tea flavor. Our results here disagree with Lim’s claim. We believe that α-farnesene could be an important compound from jasmine flowers that is infused into tea leaves during the tea process. Lin et al. [12] analyzed volatile compounds emitted from jasmine teas and found that the relative content of α-farnesene in high quality jasmine teas ranged from 14.5% to 16.51%, although linalool, benzyl acetate, (*Z*)-3-hexenyl benzonate, and methyl anthranilate were also high. As teas per se generally have rather lower concentrations of α-farnesene but higher concentrations of other compounds, such as linalool and hexenal [28], the higher α-farnesene in the quality jasmine teas documented by Lin et al. [12] lends support to our speculation that α-farnesene in jasmine teas could mainly come from jasmine flowers. It is worth noting that our study only used one cultivar, and the results were derived from one seasonal evaluation. To confirm our hypothesis, analyzing volatile compounds from different cultivars in different seasons is needed. Additionally, investigating aroma emissions from jasmine flowers, baked tea leaves, and finished jasmine teas and their changes during the scenting processes should be conducted.

This study also provides science-based information as to when flower buds should be plucked and at what stage flowers should be harvested for use in the production of jasmine teas. If flowers are harvested for immediate infusion with tea leaves locally, flowers at the stage 3 are the best choice with an option of stage 4 flowers as α-farnesene emission is higher at these stages. When flowers are harvested to be transported to factories, an 8 h time frame should be given to allow the flowers to be open at the time for infusion. The harvested flower buds should be placed in open containers at a temperature of 35 °C for flower opening. This practical guideline is based on our investigation of *J. sambac* ‘Bifoliatum’, the most popular cultivar in Fujian Province, China, and it may also be applicable to other *J. sambac* cultivars. Statistics show that less than 5% of the buds picked by experienced farmers fail to bloom that night, compared to 15% to 30% of the ones picked by inexperienced farmers [6]. In addition, experienced farmers miss less than 10% of the buds that should have been harvested while the inexperienced pickers miss as much as 20% to 30% [6]. With these guidelines, the appropriate developmental stages of jasmine flowers can be harvested for processing jasmine teas.

## 4. Materials and Methods

### 4.1. Plant Materials

*Jasminum sambac* ‘Bifoliatum’ plants grown in the plant germplasm collection garden at Fujian Agriculture and Forestry University, Fuzhou, Fujian Province, China were used in this study. This cultivar produces pure white flowers from April to August in Fuzhou. Based on the observations from flower bud appearance to flower senescence, the flowers were divided into five stages: (1) flower bud stage where all petals were close together; (2) initial-flowering stage, i.e., outside petals became loosened and flowers partially opened; (3) flowers opening stage where all petals became loosen and flowers were ready to fully open; (4) flowers fully opening stage, all petals completely opened with half of the petals becoming droopy; and (5) petals start withering (Figure 1).

### 4.2. Aroma Analysis

The Clarus SQ 8 gas chromatography-mass spectrometry (PerkinElmer, New York, NY, USA) fitted with an Elite-5MS capillary column (30 m × 0.25 mm × 0.25 μm) and Turbmatix Headspace Systems (PerkinElmer, New York, NY, USA) was used for analysis of volatile compounds.

For detecting volatiles emitted from living flowers, a single petal (each flower has 14 to 20 petals) was excised from a jasmine flower that was at one of five developmental stages in May 2015, and the flowers used for collecting petals were labeled. The collected petal was quickly weighed in the analytical laboratory (plants were grown close to the laboratory building with less than two-minute walking distance) and immediately placed into a vial fitted with a septum cap. The headspace collection vial was instantly incubated for 30 min at 60 °C with desorption time of 0.1 min and a dry purge of 5 min. Internal standard ethyl decanoate (Sigma-Aldrich St. Louis, MO, USA,) was diluted to 200 mg/L, and 100 μL diluted internal standard was added into vial. The retention indices were calculated relative to C_8_–C_20_ *n*-alkanes (Sigma-Aldrich, USA). The column temperature was held at 50 °C for 3 min, increased at rate of 8 °C/min to 160 °C, held for 2 min, then increased at rate of 3 °C/min to 180 °C, and held for 2 min. Helium was used as a carrier gas; the flow rate was 1 mL/min. The injector temperature was 230 °C in splitless mode. The Clarifi detector (PerkinElmer, New York, NY, USA) temperature was set at 240 °C. Air flow rate was 80 mL/min. MS fragmentation data from 45 to 220 amu were collected in the full-scan mode; the scan time was 0.1 s. Both the inlet line temperature and source temperature were 200 °C, and the multiplier was 1700 V. TurboMass 6.1 software (PerkinElmer, New York, NY, USA) was used. Retention indices and NIST Mass Spectral Library, including AMDIS Deconvolution, Wiley Mass Spectral Library Maurer/Pfleger/Weber Library of Drugs, Pollutants, Pesticides, and Metabolites were used for identification of the separated compounds. Based on peak areas, compound quantifications were performed relative to the ethyl decanoate. Concentrations of α-farnesene were determined in reference to a standard curve of α-farnesene (Sigma-Aldrich). The standard calibration curve was made by diluting α-farnesene into different concentrations (2–100 μg/mg) according to the GC peak area. Three flowers at each developmental stage were analyzed, and data were expressed as mean ± standard error.

For analysis of excised flowers, flower buds (stage 1) were detached from three plants and placed separately by plants at a temperature 35 °C. A single petal was excided from a randomly selected flower of each plant and used for volatile analysis at 0, 2, 4, 8, and 16 h after flower bud excision.

To investigate the effect of exogenous application of lovastatin on gene expression in the MVP pathway, lovastatin (Sigma-Aldrich) was diluted in dimethyl sulphoxide (DMSO) (Sigma-Aldrich) at 50 μm/L and sprayed on living jasmine flowers at the stage 2. Flowers at the same stage were treated with DMSO as control. Control petals or those treated with lovastatin were taken 0, 2, 4, and 6 h after application and analyzed by GC-MS as mentioned above. There were three biological replicates per treatment at each sampling time. The percent reduction was calculated for each compound.

### 4.3. Cloning Full-Length cDNA of JsHMGS, JsHMGR, JsFPPS, and JsTPS

Specific primers (Table S3) were designed according to target gene sequences of ESTs which were derived from our jasmine flower cDNA library. Total RNA was extracted from flowers using Universal Plant Total RNA Extraction Kit (Bioteke, Beijing, China). To obtain full-length coding sequence of *JsHMGS*, *JsHMGR*, *JsFPPS*, and *JsTPS* in the MVP pathway, rapid amplification of cDNA ends PCR (RACE PCR) was performed using commercial cDNA synthesis kit and ExTaq (Takara, Dalian, China) as a DNA polymerase. The first strand cDNA synthetic reaction from total RNA was prepared using the SMART^TM^ RACE cDNA amplification kit (Clontech Labs, Mountain View, CA, USA) according to the manufacturer’s instructions. RACE PCR was performed by the nest PCR method based on the instruction with the following conditions: 35 cycles at 95 °C for 40 s, 50–60 °C for 40 s, 72 °C for 100 s, and a final extension of 72 °C for 5 min. For sequencing, the PCR product was purified by GeneJET Gel Extraction Kit (Thermo Fisher Scientific, Waltham, MA, USA) and ligated into a clone vector pEASY-T (Trangene, Beijing, China). Deduced amino acid sequences were searched for homologous proteins using the NCBI databases. ClustalX with default gap penalties was used to perform multiple alignments. A phylogenetic tree was constructed by the neighbor-joining method, and the reliability of each node was established by bootstrap methods with 1000 replicates using MEGA6 software (The Biodesign Institute, Tempe, AZ, USA).

### 4.4. Quantitative Real-Time PCR Analysis

Total RNAs were extracted from the remaining three petals of flowers that were used for aroma analysis, i.e., those flowers either at different developmental stages, different times after excision, or different times after lovastatin treatment, of which 500 ng total RNAs were reverse-transcribed into cDNA by using TransScript II One-Step gDNA Removal and cDNA Synthesis SuperMix (Trangene, Beijing, China). Actin was applied as reference gene; the real-time PCR was performed in four replicates on a CFX96 Touch™ Real-Time PCR detection system (Bio-Rad, Hercules, CA, USA). The relative gene expression was calculated based on the 2^−ΔΔCt^ method [29] using actin as an internal control.

## Figures and Tables

**Table 1:** Volatile organic compounds emitted from living flowers of *Jasminum sambac* ‘Bifoliatum’ plants from stage 1 (the bud stage: petals close together) to stage 5 (flower started senescence).

Chemical Compound	Retention Index	Stage 1	Stage 2	Stage 3	Stage 4	Stage 5
		(mg/kg FW)				
Methyl acetate	986	0.20 ± 0.01 ^z^	1.80 ± 0.30	3.43 ± 1.33	2.40 ± 0.14	2.63 ± 0.76
(*Z*)-3-Hexenyl acetate	1008	0.22 ± 0.05	1.88 ± 0.43	0.79 ± 0.08	0.34 ± 0.12	0.39 ± 0.13
α-Ocimene	1016	0.02 ± 0.00	0.48 ± 0.15	0.86 ± 0.21	0.38 ± 0.02	0.21 ± 0.02
Methyl benzoate	1095	0.01 ± 0.00	0.36 ± 0.15	1.56 ± 0.78	1.97 ± 0.10	1.09 ± 0.51
Linalool	1107	3.89 ± 0.82	41.86 ± 4.99	22.93 ± 5.06	21.53 ± 1.05	8.38 ± 0.40
Benzyl acetate	1167	0.03 ± 0.00	4.66 ± 1.93	14.30 ± 2.83	10.56 ± 3.15	6.01 ± 0.12
Methyl salicylate	1193	0.03 ± 0.00	0.42 ± 0.12	1.58 ± 1.10	1.64 ± 0.18	0.54 ± 0.15
Elemene	1336	0.00 ± 0.00	0.61 ± 0.07	1.00 ± 0.19	0.90 ± 0.10	0.36 ± 0.05
Ethyl decanoate ^y^	1395	0.99 ±0.01	1.00 ±0.00	1.00 ±0.01	1.00 ±0.00	0.99 ±0.00
Caryophyllene	1420	0.02 ± 0.00	0.74 ± 0.13	0.64 ± 0.13	0.53 ± 0.13	0.22 ± 0.05
Humulene	1469	0.01 ± 0.00	0.41 ± 0.09	0.43 ± 0.05	0.33 ± 0.13	0.18 ± 0.05
(*E*,*E*)-α-Farnesene	1494	0.61 ± 0.02	12.08 ± 1.66	53.86 ± 3.49	39.03 ± 1.89	20.71 ± 4.20
Muurolene	1502	0.08 ± 0.00	1.10 ± 0.18	1.57 ± 0.25	0.93 ± 0.22	1.23 ± 0.20
(*Z*)-3-Hexenyl benzoate	1585	0.08 ± 0.05	0.45 ± 0.06	2.07 ± 0.91	1.90 ± 0.80	0.98 ± 0.13

^z^ Compounds were analyzed using GC-MS, the concentrations were determined relative to the internal control of ^y^ ethyl decanoate except for α-farnesene which was calculated based on standard curve, and data were expressed as mean ± standard error (three replications).

**Table 2:** Volatile compounds emitted from excised flowers of *Jasminum sambac* ‘Bifoliatum’ from the time of excision to 16 h thereafter at a temperature of 35 °C.

Chemical Compound	Retention Index	0 h	2 h	4 h	8 h	16 h
		(mg/kg FW)				
Methyl acetate	986	0.30 ± 0.07 ^z^	0.86 ± 0.09	1.02 ± 0.06	1.73 ± 0.05	1.06 ± 0.31
(*Z*)-3-Hexenyl acetate	1008	0.07 ± 0.01	0.97 ± 0.10	1.88 ± 0.10	0.40 ± 0.04	0.60 ± 0.12
α-Ocimene	1016	0.00 ± 0.00	0.15 ± 0.02	0.10 ± 0.01	1.13 ± 0.02	1.04 ± 0.17
Methyl benzoate	1095	0.00 ± 0.00	0.02 ± 0.00	0.22 ± 0.01	2.25 ± 0.06	0.66 ± 0.15
Linalool	1107	0.68 ± 0.05	15.76 ± 1.66	19.52 ± 2.69	22.03 ± 1.98	19.31 ± 4.34
Benzyl acetate	1167	0.05 ± 0.01 ^z^	0.09 ± 0.05	3.69 ± 0.23	16.68 ± 2.40	12.66 ± 1.76
Methyl salicylate	1193	0.01 ± 0.00	0.11 ± 0.02	0.41 ± 0.10	0.77 ± 0.19	0.57 ± 0.13
Elemene	1336	0.01 ± 0.00	0.04 ± 0.00	0.13 ± 0.05	0.25 ± 0.03	1.15 ± 0.21
Ethyl decanoate ^y^	1395	1.00 ±0.01	1.00 ±0.00	1.00 ±0.01	1.01 ±0.00	0.99 ±0.00
Caryophyllene	1420	0.00 ± 0.00	0.05 ± 0.00	0.19 ± 0.06	0.27 ± 0.03	0.23 ± 0.04
Humulene	1469	0.00 ± 0.00	0.04 ± 0.00	0.16 ± 0.02	0.28 ± 0.02	0.26 ± 0.02
(*E*,*E*)-α-Farnesene	1494	0.66 ± 0.24	1.45 ± 0.37	12.20 ± 3.02	30.66 ± 4.42	30.71 ± 0.55
Muurolene	1502	0.26 ± 0.01	0.23 ± 0.03	0.38 ± 0.11	0.47 ± 0.04	1.22 ± 0.12
(*Z*)-3-Hexenyl benzoate	1585	0.18 ± 0.01	0.63 ± 0.13	0.83 ± 0.06	1.82 ± 0.44	2.78 ± 0.34

^z^ Compounds were analyzed using GC-MS, the concentrations were determined relative to the internal control of ^y^ ethyl decanoate except for α-farnesene which was calculated based on standard curve, and data were expressed as mean ± standard error (three replications).

## Supplementary Materials

Supplementary materials are available online.

## Abbreviations

HMGS	3-Hydroxy-3-methylglutaryl coenzyme A synthase
HMGR	3-Hydroxy-3-methylglutaryl coenzyme A reductase
FPPS	Farnesyl pyrophosphate synthase
TPS	Terpene synthase
