# Supplementary material for: Volatiles Emitted at Different Flowering Stages of Jasminum sambac and Expression of Genes Related to α-Farnesene Biosynthesis
Source: Molecules. 2017 Mar 29;22(4):546. doi: 10.3390/molecules22040546 (PMC6154332; doi:10.3390/molecules22040546)
Supplement: Supplementary file 1 [file molecules-22-00546-s001.pdf]

Supplemental data_Jasmine flowers: Volatiles Emitted at Different Flowering Stages of Jasminum sambac and Expression of Genes Related to α-Farnesene Biosynthesis

Ying Yu, Shiheng Lyu, Dan Chen, Yi Lin, Jianjun Chen, Guixin Chen, and Naixing Ye

Table S1. The full-length cDNA sequence of *JsHMGS* isolated from flowers of *J. sambac* 'Bifoliatum'.

1	AAAAACTCCA GGAAATTCGC AGATCGGCCA TCGCCTACAC AGTTTCTCTC TCCGTCCCTC
61	TGCGAATTTC CTGGAGTTTT TAGATCCTTT TTGCTCGATC TGCTTTTTGC GCTTGTAAAG
121	CTAAACAAGG AAGGAGAAAG AATGGCTTCA CAAGCGAAGA ATGTTGGGAT TCTCGCCATC
181	GAAATCTACT TCCCTCCCAC TTGCATCCAA CAGGAAAAGT TGGAGGCTCA TGATGGAGCA
241	AGCAAGGGGA AATACACAAT TGGGCTTGGC CAGGATTGCA TGGGCTTTTG TACGGAGGTT
301	GAAGATGTAA TTTCCATGAG TTTGACAGCT GTTAGTTCAC TTCTTGAGAA GTATGGAGTT
361	GATCCAAAGC AGATTGGTCG TCTGGAAGTT GGAAGTGAGA CTGTACTAGA CAAGAGCAAG
421	TCTATAAAGA CATTTTTGAT GCCAATCTTT GAGAAATGCG GAAATACTGA CATAGAAGGT
481	GTTGACTCAA CCAATGCATG CTATGGGGGA ACTGCAGCTC TCTTCAACTG TGTCAATTGG
541	GTGGAAAGCA GTTCATGGGA TGGGCGATAT GGGCTCGTTG TCTGCACAGA CAGTGCGGTC
601	TATGCAGAAG GGCCAGCCAG GCCAACTGGT GGAGCTGCAG CAATTGCCAT GCTAATAGGG
661	CCTAATGCTC CTATTGTGTT TGAAAGCAAG TTGAGGGGCA GTCATATGGC TCATGCCTAT
721	GATTTCTACA AGCCTAATCT TGCCAGCGAA TATCCAGTTG TTGATGGCAA GCTTTCTCAG
781	ACGTGCTATC TCATGGCACT TGATTCTTGC TATAAAAACT TAAGTAACAA GTACAAGAAG
841	TTGGAGGGCA AACAGTTTTC AGTTGCCAAT GCTGATTACT TTGTCTTTCA TTCTCCATAT
901	AACAAGCTTG TACAGAAGAG CTTTGCCCGA TTGGTGTTCA ACGATTTTGT AAAGAATGCC
961	AGCTACATTG ATGAAGTTGC TAAAGAAAAA CTCGCACCAT TTTCATCATT AAGCAATGAT
1021	GAAAGCTACC AAAGTCGTGA TCTTGAAAAG GCATCTCAGC AAGTTGCAAA GCCCTTTTAT
1081	GATGCCAAGG TGCAGCCATC CACATTAATT CCAAAGCAAG TCGGCAACAT GTACACTGCA
1141	TCAATCTATG CTGCATTTGC ATCTCTTATT CACAACAAGC ATAGCACACT GGCTGGGCAG
1201	AGGGTGATCT TGTTCTCCTA TGGTAGTGGT TTGAGTGCTA CCATGTTCTC AGTCCGTCTT
1261	AACGAGGGTC AACATCCTTT CAGCCTATCC AACATTGCAA CTGTCATGAA TGCTGCAGAG
1321	AAGTTGAAAT TGAGGCATGA GTTGCCTCCA GAAAAATTCG TCGAGATCAT GCAAGTAATG
1381	GAGCATAGGT ATGGGGCAAA GGACTTTGTG ACAAACAAGG ACACGAGTCT TTTAGCACCA
1441	GGCACATACT TCCTTACGCA CGTGGATTCC ATGTACCGGA GATTCTACTC CAAGAAGTCT
1501	ACTGAGAATG GCTTACTAGC CAATGGTCAT TGAAGATAGA TTGTGACGTA CCAGAAGTGA
1561	CGCTATGAAG TTAGCTCGTG TTTTTAGCTT GTAGAAGAAT AAGTTACATG TTCATTTATT
1621	GATACAAACA ATTAGTTTCT TTCAGTTCTT TCTGGTTCTT TTTTCTCTTC TATTAGCTTA
1681	TATTGTATTG GTTTTGTCAA TGACATCAAG AATTTTTTAC GCTTATTAAT AATTCGAATG
1741	ATCTTTTAGA GCTATGTTAC TTTTCCGACG TGTGTTTTGA AGTGTGTTCA AAATGTGAAC
1801	TAAACTTG

Table S2. The full-length cDNA sequence of *JsTPS* isolated from flowers of *J. sambac* 'Bifoliatum'.

1 TGGCCATTAC GGCCTAGTTA CGGGGGAGGG CATCTGCAAA CCATTCCTTA ATTTCTGTTG
61 TTAAACTACT AATAATACAT ATCAAGAATG GAGCAAAAGA ACGAGTTAGA CTCCATTGAA
121 AGGCGAAATG CTAACTATAA GCCGAACATC TGGAATTACT CTGTTTTGCA ATCTCTCACA
181 AACAAATACG ATGAAGAGAA ATACGGAAGA CAAGTGGAAA TGTTGAAAAT GGAGGTCTCG
241 TGCATATTTG AGGAAGTGAA AGATCCCTTG GCTAAATTAG AGCTTATAGA TTGCATTGGA
301 AAATTAGCTT TGTCTCACTA CTTCGAGAAA GATATATATA AAACCCTAAA CATGATGGTG
361 AAAAATTCGG ACAACTTCGT TTCTTGCATG GAAGAGAACC TCTACCTCAC CGCATTATAT
421 TTTCGAATTC TTAGGACGTA TGGATGTGAA ATTTCACAAG ACGTGTTTCT CTCCTTCACC
481 AATGGCATGG GCCAGTTCAC GACAAGCCCG GATGTGGATT CCAAACAGAT TCTTCAACTA
541 TTGGAAGCTT CTTATCATGG CATGGAAAGT GAATTCTTGT TGGACCAAGC CCAAGTTTTT
601 GCCACCAAGA ATCTGATGAT CACTGCAAAA AATAGCCTCA TTTGTCCATT GCATTTGAGT
661 GTCGAATGGT TTAACGTTAA ACGACATATT CATGCACACG AAAAAGAGAA TAAAACTAAG
721 TCTACGTTAC TTCAGCTTGG TAGGCTCAAC TTCAACATAT CTCAAGCCCA ACAACAGCAG
781 GATCTCAAGG AAATTTTAAG GTGGTGGAAC CAGCTGGACC TCTCAGAAAC CTTAACTTTC
841 ACGAGAAATC GGGTGGTGGA GAGTTTTCTG TGGTCGGTCG GGGTTGCGTC TGAAACCCAG
901 CATGCGAGCT TGCGAAAATG GCTTACGAAA GTCATCATGT TCGTCTTGAT AATTGACGAT
961 GTTTATGACA TTTATGGGTC CATGGAAGAG TTAGAATGCT TTACACGTGC TGTTAAAAGG
1021 TGGGAGTATG CGGAAGTTGA GCAGCTACCA GAAGCCATAA GAAGATGCTT CTGTGCACTA
1081 CAGGATACTA CATATGACAT TGACCATCAA ATTCAACAAG AGAAGGGCTG GGATTCAGTG
1141 TTACCTTATT TGATGAAAGG GGGGACAGAT TTATGTGGAG CCTTGCACGT GGAAGCGAAG
1201 TGGTACCACA CAGGTTATAC CCCACGACTA TGGGAGTATC TATCTAATGG TTGGATATCA
1261 TCTTCTGGTC CCCTGCTTTC CCTTGTTGTA CTCTTGGGTC TACCTGGTCG AGATGTAGCA
1321 GAAACCATAG AACTCCTCGA GGAAAATCAA GAAATTATCT ACTATGCCTC GTTGATAATA
1381 CGCCTTTGCA ATGATCAAGG AACTTCAACT GCCGAGCTGG AGAGGGGAGA CGCACCTTCG
1441 TCAATCCTGT GTTATATGAG AGAAGCAAAT GTTACAGAAG AGGAAGCTAG GGAACACATC
1501 AGAAAAATAA TTGGGAATCT ATGGAAGAAA ATCAATGGCC TATGTGTAAA AGGTTGTCCT
1561 AATTTGTTTC AAACTCCCAT TAAGCACATT GTTAACACAG CGCGAGTTGC TAATTTCATT
1621 TATCAAAAGG GAGATGGATT TAGTGTTCCA GATGGAGACA CGAAAAATCA AGTGCTTTCG
1681 TGTTTAATAG AGCCTTTTCT GCTGGTGTAA ATTTTTTTAT GAGAAGCAAA CATCATCTAA
1741 GTTAGTCGAA TAATGTACTT TTTATTGGGA GTGTAATAAA CCAGTTGATT TGGCATTTAA
1801 AATATATTTT TGCATCTTTA TTGGTGTGCA AAAATCGTAG ATTTTTCCCC TTTTTTTCCA
1861 TGGAGTATGG TACATTGGTA CATGTAATAA AGTTTGAGTT AGTCCCTATC ATACTTGGGA
1921 TGGGGGTTTA TTGCAATATA CTAGTAATTG TGTTCCAAAA AAAAAAAAAA AAAA

Table S3. Primers used for qRT-PCR analysis of for *JsHMGS*, *JsHMGR*, *JsFPPS* and *JsTPS* expression in flowers of *Jasminum sambac* 'Bifoliatum'.

Primer name	Primer Sequence(5'-3')
JsHMGS-F	AACATCCTTTCAGCCTATCC
JsHMGS-R	GGAAGTATGTGCCTGGTGCT
JsHMGR-F	CGAGGAAGATAGTCGGATGG
JsHMGR-R	CTCCTCTGACGATTGCGGTGT
JsFPPS-R	ATCTGACCCGAAGCAGTTTGG
JsFPPS-F	GGTATGATTGCGGCGAATGATG
JsTPS-R	AACCATTAGATAGATACTC
JsTPS-F	ACTACAGGATACTACATA
Actin-R	CAACCAAACCGTCTTCGCTT
Actin-F	CTATCGTCCACAGGAAATGCT

Primers were designed for qRT-PCR analysis of relative expressions of genes encoding 3-hydroxy-3-methylglutaryl coenzyme A synthase (HMGS), 3-hydroxy-3-methylglutaryl coenzyme A reductase (HMGR), farnesyl pyrophosphate synthase (FPPS), and terpene synthase (TPS) in *Jasminum sambac* flowers with actin gene as an internal control.
